# Maternal Satisfaction and Its Associated Factors towards Spinal Anesthesia for Caesarean Section: A Cross-Sectional Study in Two Eritrean Hospitals

**DOI:** 10.1155/2020/5025309

**Published:** 2020-03-21

**Authors:** Idris Mohammed Idris, Ghidey Gebreyohanns Weldegiorgis, Eyasu Habte Tesfamariam

**Affiliations:** ^1^Department of Anesthesia and Critical Care, School of Nursing, Asmara College of Health Sciences, Asmara, Eritrea; ^2^Asmara College of Health Sciences, Asmara, Eritrea; ^3^Department of Epidemiology and Biostatistics, School of Public Health, Asmara College of Health Sciences, Asmara, Eritrea

## Abstract

**Objective:**

Satisfaction of mothers during caesarean section is an important indicator for measuring quality of obstetric anesthesia. This study aimed to determine mothers' level of satisfaction and the predicting factors of dissatisfaction towards spinal anesthesia during caesarean section.

**Methods:**

Cross-sectional study design was utilized in Orotta Maternity Hospital (OMH) and Sembel Hospital from December 2017 to February 2018, in Asmara, Eritrea. Satisfaction of the mothers was measured using a pretested questionnaire. Bivariate and multivariate logistic regression were utilized to identify predictors of dissatisfaction using SPSS (Version 22.0).

**Results:**

Involvement of mothers in the choice of anesthesia (3.3%) and explanation about the stay at operating theater (10%) were the two least reported items. As per the subscale analysis, the lowest satisfaction was observed for the preoperative assessment (16.7%). Overall, 87.9% of the mothers were satisfied with the spinal anesthetic service. Hospital at which anesthesia was administered (*p* < 0.001), marital status (*p* < 0.001), and intraoperative pain (*p* < 0.001) were significant predictors of dissatisfaction towards spinal anesthesia. Moreover, the rate of refusal to have spinal anesthesia in the future was 12.5%.

**Conclusion:**

Though overall satisfaction can be considered as fair, preoperative assessment is considerably low. Hence, explaining the benefits and risks of the anesthetic techniques as well as considering patient's opinion is very important while deciding the type of anesthesia.

## 1. Introduction

Satisfaction with service delivery is one of the major determinants of quality of care in a hospital [1]. Globally, out of 213 million deliveries, caesarean section (CS) accounted for 18.5 million indicating its increased rate [2]. In the last two decades, the rate of CS has tripled [3]. Likewise, the rate of CS is increasing in Eritrea. For instance, Orotta Maternity Hospital (OMH) indicated a CS of 13.3%, whereas Sembel Hospital reported 35.3% [4]. Letting the mother awake, minimal depression of fetus, small drug dose, low failure rate, rapid onset, and simplicity of its technique make spinal anesthesia more popular than general anesthesia for CS [5]. Moreover better neonatal outcome was recorded for newborns from mothers who underwent regional anesthesia compared to general anesthesia for their CS [6]. Since 2001, more CS were being done under spinal anesthesia [7], but some countries reported higher rates of general anesthesia for CS [3]. Similarly spinal anesthesia (SA) for caesarean section has become the routine and preferred technique among Eritrean anesthetists. Anesthesia is administered by the so called “nurse anesthetists” in Eritrea, because there are no anesthesiologists. With increasing rate of CS, the demand for SA has consequently increased. Therefore ensuring the quality of obstetric anesthesia becomes a vital concern which the anesthetist needs to put emphasis on. Though SA is preferred by majority of anesthetists, it was quite important to see whether the patient population was as appreciative. Assessing maternal satisfaction towards spinal anesthesia during caesarean section could be an important aspect for measuring the quality of anesthetic care. Identifying factors that promote or compromise maternal satisfaction will help in the development of protocols in the services of obstetric anesthesia. Research studies conducted on maternal satisfaction towards spinal anesthesia for CS revealed variations. Some developed countries reported higher satisfaction rate [8] whereas in the developing countries, such as Ethiopia, maternal satisfaction was slightly lower, reaching 62% [9]. In Eritrea there has been no known study addressing the subject. This study was therefore conducted to determine mothers' level of satisfaction and the predictors of dissatisfaction towards spinal anesthesia during caesarean section. Furthermore, it addressed the potential refusal of spinal anesthesia and the related reasons.

## 2. Methods

### 2.1. Study Design and Setting

An analytical and cross-sectional hospital-based study with a quantitative approach was conducted in two selected hospitals (namely, Orotta Maternity National Referral Hospital (OMNRH) and Sembel Hospital). Data were collected from December 2017 to February 2018 in Asmara, Eritrea. OMNRH is the largest national referral hospital providing comprehensive maternal services. It is a public hospital. Not only does it provide health services but it also functions as a teaching hospital where medical and nursing students practice their clinical sessions, having 5 anesthetists. As per the 2017 records, it carries out a total of about 8047 deliveries per year, out of which 13.3% (1068) were through caesarean section. Sembel Hospital is the only private hospital in Eritrea which provides integrated medical services including maternal service, having 3 anesthetists. According to 2017 hospital record, it had 1191 total deliveries, out of which 35.3% were through caesarean section.

### 2.2. Source and Study Population

All subjects who underwent caesarean section from December 2017 to February 2018 were the source population for this study. Subjects who underwent caesarean section through the spinal anesthetic technique only and for whom less than 24 hours had elapsed from the time of delivery formed the study population. Subjects who underwent general anesthesia after failure of spinal anesthesia (SA), those who underwent other modes of anesthesia (e.g., general or epidural anesthesia), and those who were unconscious after surgery and unwilling to participate were excluded from the study. However all the study populations within the specified timeline were included starting from data collection to analysis (census method).

### 2.3. Variable Measurements

The dependent variable used in this study is maternal satisfaction towards spinal anesthesia. The independent variables used in this study are sociodemographic variables and factors related to surgery, anesthesia, and disease status.

Sociodemographic variables include age, educational status, marital status, employment status, and parity. Factors related to surgery, anesthesia, and disease status include previous experience of surgery and anesthesia, past medical illness, and perioperative factors.

### 2.4. Data Collection Tool

A structured and pretested questionnaire was used to collect information. The data collection tool consisted of thematic areas that include sociodemographic information of mothers, previous medical and anesthesia history, and perioperative data which addresses mothers' satisfaction and a standard tool which measures the level of satisfaction of the mothers. The standard tool was adopted from Patient Satisfaction with Perioperative Anesthetic Care questionnaire (PSPACq) with slight modification [10]. The modifications were done as per suggestion of the panel of experts with reference to the study setting and cultural concerns. Upon modification, items that were found to focus on general anesthesia were excluded because the main concern of the study was spinal anesthesia. Moreover, the five-point Likert scale was modified to three-point Likert scale due to inability of the mothers to distinguish “slightly satisfied” and “very (completely) satisfied.” The final tool that the researchers utilized consisted of 25 items with six subscales. The three-point Likert scale was scored as 1: dissatisfied, 2: neutral, and 3: satisfied. The first subscale, “preoperative assessment and evaluation,” measures the level of satisfaction with regard to preoperative evaluation. The second subscale, “pain therapy,” measures the level of satisfaction of the mothers with regard to the pain they exhibit during and after the anesthetic procedure. The third subscale, “attention by anesthetist,” measures the level of satisfaction with regard to the attention given to them by the anesthetist during intraoperative time. The fourth subscale, “anesthetist-patient relationship,” measures the level of satisfaction with regard to the anesthetist interpersonal relationship with the mothers. The fifth subscale, “postoperative care,” measures the level of satisfaction with respect to the postoperative care given to them. The sixth subscale, “quality care,” measures the level of satisfaction with respect to the quality of care given to them.

The original English version questionnaire was first translated into Tigrigna by one person and then back-translated into English by another one.

### 2.5. Preliminary Reliability and Validity Assessments

Exploratory factor analysis with interitem correlation and item discriminant validity was computed to assess the factor loading structure of the questionnaire. The overall internal consistency of the scale used was computed and was found to be satisfactory (Cronbach-*α* = 0.612). Cronbach's alpha for the six subscales ranged from 0.581 to 0.726. Face and content validity of the questionnaire was determined through the review of experts in various relevant fields.

### 2.6. Data Collection Procedure

Training was given to the data collectors before the start of data collection. The data collector was approaching the potential participants within six to twenty-four hours of the postoperation period. The trained data collector was explaining the aim of the study to the participants, and those who signed the written informed consent were recruited in the study. Then face-to-face interview was conducted. All activities in data collection were supervised by the principal investigator.

### 2.7. Statistical Analysis

Data was entered and analyzed using SPSS software version 22. Before entering data, the questionnaire was checked for its completeness and consistency by the principal investigator and trained data encoders. Overall satisfaction of mothers was measured for all the six subscales: preoperative assessment and evaluation (7 items), pain therapy/control (3 items), attention by anesthetist (4 items), anesthetist-patient relationship (4 items), postoperative care (2 items), and quality of care (5 items). The three-point Likert scale responses of satisfaction were summed up and dichotomized into “dissatisfied” and “satisfied” for analysis. The panel of experts, after consideration of the setup and similar study in Ethiopia [9], decided that satisfaction of 50% and above is considered as “*satisfied*.” Descriptive statistics were used to summarize and show frequency distribution and percentages of the variables. M ± SD was used for a continuous variable and percentages for categorical variables. Furthermore, bivariate and multivariate logistic regression analyses were used to determine the strength and direction of association at 0.05 significance level.

### 2.8. Ethical Approval

Ethical clearance was obtained from ACHS and Ministry of Health Scientific and Research Ethical Committee. After securing permission from ACHS and the MOH, letter of support was sent to both OMNRH and Sembel Hospital for allowing data collection. Informed consent was obtained from each respondent after a thorough explanation of the aim and potential benefits of participating in the study, and written consent was signed by the mothers. Anonymity and confidentiality were ensured in that the respondent's name never appeared on the questionnaire.

## 3. Results

### 3.1. Sociodemographic and Background Characteristics

A total of 240 respondents with a mean age of 30.2 (SD: 5.4) were enrolled in the study. Majority of the respondents were from Orotta Maternal Hospital and 29.6% were from Sembel Hospital. Only 17 (7.1%) were single and about 70% had attained secondary and above educational status (Table 1).

### 3.2. Parturition History

More than half (56.3%) of the respondents had undergone elective surgery and the remaining 43.7% were emergency cases (Table 2). The majority of the respondents were multiparous (68.3%). Most of the neonates were born in good condition with 151 (62.9%) having birth weight of greater than or equal to three kilograms. Two hundred newborns (83.3%) had an Apgar score of 10 at 5 minutes.

### 3.3. Past Medical and Anesthetic History

The majority of the respondents were in a good health condition with only 11 (4.6%) having a known medical condition (Table 3). More than half of the mothers (53.3%) had received anesthesia for prior CS and about forty-six percent of them through spinal anesthetic technique. Of the total respondents, only 21 (8.8%) mothers had developed complication from the previous experience of surgery and anesthesia, with pain being the leading complication followed by backache, whereas postoperative nausea and vomiting (PONV) was a rare complication.

### 3.4. Satisfaction of Mothers towards Anesthetic Service

#### 3.4.1. Preoperative Services

More than 80% of the mothers were not satisfied with preoperative information given by anesthetists about their feeling after taking anesthesia, the average stay in the operating theater, and the progress and complication of surgery. Even though the mothers had good understanding of the little preoperative information, the majority (69.2%) of them were not satisfied with preoperative visit of the anesthetist (Table 4).

#### 3.4.2. Pain Therapy

The majority of the mothers (86.3%) were satisfied with the absence of pain during lumbar puncture, and almost all (96%) were satisfied with pain control during operation, but pain therapy after surgery was not satisfactory accounting for 25% of mothers dissatisfaction.

#### 3.4.3. Intraoperative Care and Attention of the Anesthesia Provider

Almost all of the mothers were satisfied with the intraoperative care of the anesthetists with more than 94% of them being satisfied with the attention of the anesthesia providers to their complaints, their sympathy, and willingness to listen to their questions and needs.

#### 3.4.4. Anesthetist-Patient Relationship

The majority (91.7%) of the mothers were satisfied with politeness of the anesthetists and the consideration of their cultural/ethnic background. However, involvement of mothers to decide what type of anesthesia they receive was very low such that 96.3% had dissatisfaction due to the lack of self-involvement for the type of anesthesia they received.

#### 3.4.5. Postoperative Follow-Up of the Mothers by an Anesthetist

About 94% of the mothers showed satisfaction with the treatment of nausea and vomiting after surgery, but 75.8% of them had dissatisfaction due to the lack of postoperative follow-up/visit of the anesthetist.

#### 3.4.6. Quality of the Care

Of the total respondents, more than 85% were satisfied with the quality of the anesthetists' care including their practice, professionalism, and skill. Thus they would not hesitate to recommend them for themselves or their family members.

### 3.5. Subscale and Overall Satisfaction of Mothers

**Figure 1:**
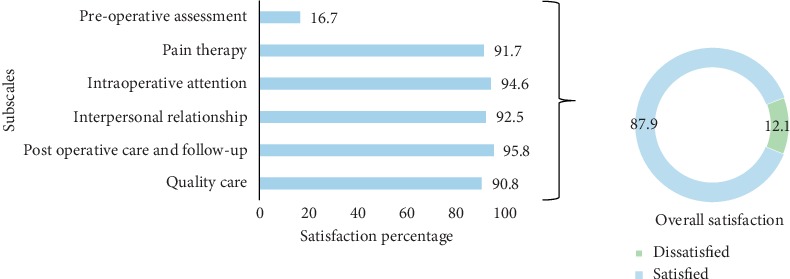
Overall and subscale satisfaction of mothers towards anesthetic services of spinal anesthesia, Asmara, Eritrea, 2018.

The respondents had an overall satisfaction of 87.9% towards anesthetic services of spinal anesthesia (Figure 1). Subscale-wise, the majority of patients (94.6%, *n* = 227) were satisfied with getting attention from the anesthetists, followed by anesthetist-patient interpersonal relationship (92.5%, *n* = 222), pain therapy (91.7%, *n* = 220), and preoperative assessment and evaluation (16.7%, *n* = 40).

### 3.6. Factors Associated with Maternal Dissatisfaction

Hospital where the mothers gave birth through CS and marital status were significantly associated with maternal dissatisfaction (Table 5). Mothers from Sembel Hospital were 5.35 times more dissatisfied than those from Orotta (AOR = 5.35, 95% CI = 2.16, 13.27). Unmarried mothers were almost 10 times more dissatisfied than the married ones (AOR = 9.79, 95% CI = 2.88, 33.37).

Among the past history and perioperative variables, presence of intraoperative pain was the only variable significantly associated with maternal dissatisfaction. Mothers who had intraoperative pain were about 11 times more dissatisfied than those who did not have intraoperative pain (AOR = 11.19, 95% CI = 2.03, 61.75).

### 3.7. Refusal Rate of Future Spinal Anesthesia

**Figure 2:**
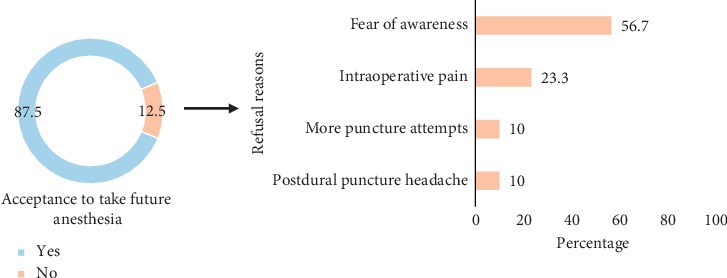
Acceptance to undergo future spinal anesthesia by the participants, Asmara, Eritrea, 2018.

Out of the total 240 mothers, 210 (87.5%) would choose spinal anesthesia again, whereas 12.5% would refuse to undergo spinal anesthesia in the future (Figure 2). Fear of awareness during operation was the leading reason (56.7%) for mothers' refusal of future SA followed by intraoperative pain (23.3%), several puncture attempts (10%), and postdural puncture headache (10%).

Dissatisfaction of mothers was found to be a significant predictor of refusal to future spinal anesthesia (OR = 5.03, 95% CI: 2.06, 12.28, *p* < 0.001).

## 4. Discussion

Researching patient satisfaction is important as it helps us in improving the quality of anesthesia [8]. Several aspects of perioperative care that were thought to likely influence maternal satisfaction were assessed. The overall satisfaction rate of mothers towards spinal anesthetic service for caesarean section was 87.9%. It was fairly good as compared to Ethiopian (62%) [9] and Kenyan studies (85%) [11], but it is still lower compared to a Malaysian study [8] which reported satisfaction rate of 97%. The difference could be attributed to setup and capacity differences between the countries. Comparing the two hospitals, we found that mothers delivered in Sembel Hospital were significantly more dissatisfied than those from Orotta. Sembel Hospital is the only private hospital in Eritrea. Hence, patients served in the hospital are those having high socioeconomic status and higher educational level. The fact that mothers pay more in Sembel Hospital may have raised the rate of dissatisfaction. Additionally, the expectations of patients are higher from a private hospital than a public hospital. The significant difference could be therefore due to the mismatch between their expectations and the poor service.

A number of studies have identified poor preoperative communication as a significant negative predictor of maternal satisfaction [12]. Similarly in our study a very low satisfaction of mothers with preoperative assessment was reported, accounting for 16.7% of satisfaction rate. Satisfaction with preanesthesia information about the procedure was low (36%) in a study done in Kisumu County Hospital [11]. The lack of adequate anesthetists may be the probable cause for not providing sufficient information. In our setting, airway problems in the acute and intensive care departments are managed by the few anesthetists on the top of surgical patients. Postoperative visit or follow-up was also among the ignored aspects, with 75.8% of mothers being dissatisfied due to the lack of anesthetist visit or follow-up after surgery. In this study mothers were given metoclopramide as an antiemetic prophylaxis, and most patients were punctured with a small gauged spinal needle (25G) which probably had decreased the incidence of PONV and PDPH, respectively. However satisfaction of mothers was high for the attention and anesthetists interpersonal communication during the intraoperative time. These findings clearly indicate the neglecting of preoperative and postoperative practices of clinical anesthesia in both the hospitals. Lack of preoperative information is a problem in many developing countries including Eritrea. Beyond its psychological importance to mothers, preoperative assessment is an indication of professional competency and quality of anesthetic care. Preoperative anesthesia and intraoperative anesthesia have equal importance, and it is the responsibility of the anesthetist to make preoperative assessment and evaluation. Lack of preoperative communication and evaluation makes mothers lose confidence, i.e., affects their satisfaction negatively. Anesthetists need to extend their practice in preoperative and postoperative care rather than being restricted only in intraoperative management. Hence, the regulatory bodies and health training institutions need to increase the number of trained anesthetists. The level of mothers' satisfaction with deciding the type of anesthesia they received was also extremely low (3.3%); i.e., 96.7% were dissatisfied, which indicates that mothers' freedom of choice was ignored. From ethical and legal point of view, any patient has the right to choose the care he/she receives; however, shortage of time on one side and burden of patients on the other side make anesthetists administer the preferred technique without considering the mothers' choice.

Intraoperative pain assessment is an important factor for excluding block failure. Many anesthesia providers claim spinal anesthesia to have a complete analgesia during intraoperative and immediate postoperative time, but still failure may happen. There was a high level of maternal satisfaction with pain control. Similar results of pain control were reported from a Kenyan study [1]; though very few studies on intraoperative pain have been done, with most studies concentrating on postoperative pain, intraoperative pain control in Pakistan [13] showed a satisfaction score of 74.08%. The difference could probably be due to the timing of data collection. The study done in Pakistan had no limit on time of data collection after delivery. Therefore, delayed adverse effects of spinal anesthesia such as postdural puncture headache were more likely to have occurred in the Pakistan study. The significance of this finding indicates that spinal anesthesia especially with bupivacaine is effective in controlling pain during and immediately after surgery.

There was a significant relationship between marital status and mother's dissatisfaction. Single mothers were more dissatisfied than the married ones. Another study [11] reported comparable results. Significant dissatisfaction of single mothers is an area of exploration as there might be a higher rate of unwanted or unexpected pregnancies. Literature findings indicated that the difference in awareness between higher and lower educated mothers towards health services affects their satisfaction due to the high expectation of patients with relatively higher education. For example, in a study conducted at Jimma Hospital, dissatisfaction increased with increase in educational level but decreased with increase in age of respondents [9, 14]. Contrastingly, our results indicated no statistical significance regarding the relationship between levels of mothers' education and satisfaction. The crude odds ratio of mothers' age and parity showed significance in the univariate analysis; i.e., the younger the mothers, the lower the satisfaction rate. These variables could also attain statistical significance after adjustment if larger sample is used. The number of puncture attempts, neonatal birth weight, and their Apgar score did not have statistical significance in influencing the overall satisfaction. Apgar scores recorded by an experienced neonatologist for every newborn are part of good clinical practice. In an international survey by Harazim et al. [6], an experienced neonatologist was recording the Apgar score; thus a cut-off of seven and below was considered as poor outcome. In our case Apgar score below seven is considered as life-threatening. As our hospitals did not have neonatal ICU and neonatologists, newborns were resuscitated by midwives and nurse anesthetists within the operating theater. Hence, an Apgar score of nine and below was considered as bad outcome in our setting. In the study conducted by Harazim et al. [6], outcomes of newborns born from mothers who underwent regional anesthesia for CS had significantly better Apgar scores compared to those who underwent general anesthesia. Our study did not compare spinal anesthesia with general anesthesia; this is a gap for future research in Eritrea.

Experiencing intraoperative pain had significant influence on maternal dissatisfaction. Though spinal anesthesia is expected to block pain sensation, some patients may experience pain due to either inadequate anesthesia or patients' psychological perception differences. In this study, about five percent of the mothers were experiencing pain and all of them were dissatisfied; this indicates the high impact of pain on mothers' satisfaction. We did not utilize any pain assessment tool as our aim was not to assess the pain level. They were simply asked whether they were feeling pain or not. There was better satisfaction with management of postoperative nausea and vomiting (PONV) and postdural puncture headache (PDPH). The fact that they were given metoclopramide as an antiemetic prophylaxis and most patients were punctured with a small gauged spinal needle (25G) could probably had decreased the dissatisfaction towards PONV and PDPH.

Refusal of taking future spinal anesthesia could be a negative indicator of satisfaction with the current experience of spinal anesthesia. This study revealed a refusal rate of 12.5% for future spinal anesthesia. Fear of awareness during operation was the leading reason (7.1%) making mothers refuse future SA, followed by intraoperative pain. Other studies found variable refusal rates. Our study revealed a lower refusal rate compared to the studies done in Greece 20% [15], Czech Republic 30% [3], and Ethiopia 18% [9]. Headache and backache were the main reasons of refusal in the Ethiopian study. Similarly most antenatal mothers in Nigeria preferred general anesthesia in the future [16]. Eight percent (8%) of mothers refused the option of receiving future spinal anesthesia due to awareness and failed regional anesthesia [8]. From the above compared studies, it can be deduced that the reasons of refusing future spinal anesthesia are quite similar. Our study also showed significant influence of dissatisfaction on refusal of future SA. Such a refusal can be minimized by explaining the advantages of SA over GA.

### 4.1. Limitation of the Study

As patients like to please service providers by replying “satisfied,” there could be risk of overestimating the satisfaction level. The study was conducted only in Asmara, and hence, results may not be subject to extrapolation to that of the country.

## 5. Conclusion

In general, the overall satisfaction of the mothers towards spinal anesthesia was fair. Relatively, the mothers were highly dissatisfied on preoperative assessment subscale as compared to the other five subscales. Risk factors contributing to dissatisfaction were hospital at which the anesthesia was administered, marital status, and intraoperative pain. Fear of awareness, pain, several puncture attempts, and postural puncture headache were the main reasons of refusal to have spinal anesthesia again. Explaining the benefits and risks of the anesthetic techniques as well as considering patient's opinion is very important when deciding about the type of anesthesia.

## Figures and Tables

**Table 1:** Sociodemographic and background characteristics of the study participants, Asmara, Eritrea, 2018.

Variables	Frequency	Percent (%)
Age (mean = 30.2, SD = 5.4)		
18 to 26	65	27.1
27 to 35	119	49.6
36 to 42	56	23.3

Hospital		
Sembel	71	29.6
Orotta	169	70.4

Level of education		
Junior or below	73	30.4
Secondary or above	167	69.6

Employment		
Employed	55	22.9
Unemployed	185	77.1

Marital status		
Married	223	92.9
Single	17	7.1

**Table 2:** Parturition history and outcomes of the participants, Asmara, Eritrea, 2018.

Variables	Frequency	Percent (%)
Parity		
Primipara	76	31.7
Multipara	164	68.3

Newborn birth weight (Kg)		
Less than 3	89	37.1
3 or more	151	62.9

APGAR score at 5 minutes		
<10	40	16.7
10	200	83.3

Nature of surgery		
Elective	135	56.3
Emergency	105	43.8

**Table 3:** Past medical and anesthetic history of the study participants, Asmara, Eritrea, 2018.

Variable	Frequency	Percent (%)
Any medical history		
Yes	11	4.6
No	229	95.4

Anesthesia history		
Yes	128	53.3
No	112	46.7

Received anesthetic technique		
GA	18	7.5
SA	110	45.8

Any complication after anesthesia		
Yes	21	8.8
No	107	44.6

Kind of complication developed		
PONV	2	0.8
Pain	14	5.8
Backache	5	2.1

PONV: postoperative nausea and vomiting; GA: general anesthesia; SA: spinal anesthesia.

**Table 4:** Satisfaction level of mothers towards anesthetic service item-wise, Asmara, Eritrea, 2018.

Satisfaction subscales	Level of satisfaction of the mothers		
	Dissatisfied *N* (%)	Neutral *N* (%)	Satisfied *N* (%)
Preoperative assessment and evaluation			
Satisfaction with the amount of information given from the anesthesia practitioners about anesthesia	193 (80.4)	9 (3.8)	38 (15.8)
Explanation about operation	77 (32.1)	20 (8.3)	143 (59.6)
Explanation about your stay at operating theater	211 (87.9)	5 (2.1)	24 (10)
Understandable information	29 (12.1)	15 (6.3)	196 (81.7)
Sufficient explanation about the feeling after anesthesia	195 (81.3)	7 (2.9)	38 (15.8)
Satisfaction with preoperative visit	166 (69.2)	37 (15.4)	37 (15.4)
Satisfaction with information about postoperative complication	204 (85)	13 (5.4)	23 (9.6)

Pain therapy			
Satisfaction with postoperative pain	28 (11.7)	5 (2.1)	207 (86.3)
Satisfaction with the absence of pain at puncture site	10 (4.2)	0 (0)	230 (95.8)
Satisfaction with absence of pain during operation	60 (25)	11 (4.6)	169 (70.4)

Intraoperative care and attention of the anesthetist			
Satisfaction of anesthetist's attention to your complaints like pain and nausea	7 (2.9)	7 (2.9)	226 (94.2)
Satisfaction with degree of anesthetist's will to listen your questions	6 (2.5)	8 (3.3)	226 (94.2)
Action according to your needs	8 (3.3)	5 (2.1)	227 (94.6)
Anesthetist showing understanding of your situation	9 (3.8)	5 (2.1)	226 (94.2)

Anesthetist-patient relationship			
Did the anesthetist take your privacy into account?	121 (50.4)	16 (6.7)	103 (42.9)
Anesthetist's respect/politeness	15 (6.3)	5 (2.1)	220 (91.7)
Did the anesthetist take your cultural background into account?	10 (4.2)	1 (0.4)	229 (95.4)
Satisfaction with the chance for your decision on type of anesthesia received	231 (96.3)	1 (0.4)	8 (3.3)

Postoperative care and follow-up of mothers by the anesthetist			
Satisfaction with anesthetist's postoperative visit	182 (75.8)	15 (6.3)	43 (17.9)
Satisfaction with PONV treatment	10 (4.2)	4 (1.7)	226 (94.2)

Quality care			
Satisfaction with waiting time between your arrival at theater and operation	31 (12.9)	12 (5)	197 (82.1)
Satisfaction with receiving the same anesthetic again	18 (7.5)	14 (5.8)	208 (86.7)
Degree of your confidence in the anesthesia practitioners	4 (1.7)	24 (10)	212 (88.3)
Degree of professionalism of your anesthesia practitioners	9 (3.8)	13 (5.4)	218 (90.8)
Recommendation of the anesthesia team to others in your family	27 (11.3)	6 (2.5)	207 (86.3)

PONV: postoperative nausea and vomiting.

**Table 5:** Predictors of maternal dissatisfaction using bivariate and multivariate logistic regression, Asmara, Eritrea, 2018.

Variable	COR	95% CI	AOR	95% CI
Hospital				
Sembel	**5.81** ^*∗∗∗*^	2.54, 13.29	**5.35** ^*∗∗∗*^	2.16, 13.27
Orotta	1			

Age				
18 to 26	**2.99** ^*∗*^	1.25, 7.19	2.92	0.60, 14.23
27 to 35	2.8	0.94, 8.35	1.48	0.42, 5.18
36 to 42	1			

Educational level				
Secondary or above	1.03	0.45, 2.39		
Junior or below	1			

Employment				
Employed	0.86	0.33, 2.24		
Unemployed	1			

Marital status				
Single	**15.34** ^*∗∗∗*^	5.24, 44.91	**9.79** ^*∗∗∗*^	2.88, 33.37
Married	1			

Surgery				
Emergency	1.05	0.48, 2.29		
Elective	1			

Parity				
Primipara	**2.24** ^*∗*^	1.02, 4.92	0.94	0.26, 3.39
Multipara	1			

Neonatal birth weight (Kg)				
Less than 3	1.98	0.91, 4.33		
3 or more	1			

Apgar score				
<10	1.05	0.37, 2.93		
10	1			

Medical illness				
Yes	0.72	0.09, 5.82		
No	1			

Anesthesia history				
Yes	0.79	0.37, 1.73		
No	1			

Prior anesthesia				
SA	0.56	0.14, 2.22		
GA	1			

Intraoperative pain				
Yes	**23.29** ^*∗∗∗*^	6.58, 82.43	**11.19** ^*∗∗∗*^	2.03, 61.75
No	1			

PDPH				
Yes	0.37	0.09, 1.64		
No	1			

PONV				
Yes	0.72	0.09, 5.82		
No	1			

Puncture attempts				
>2 trials	2.29	0.64, 7.67		
<=2 trials	1			

1: reference category; ^*∗*^, ^*∗∗*^, ^*∗∗∗*^ significant at 0.05, 0.01, and 0.001 level, respectively; OR: odds ratio; AOR: adjusted odds ratio; CI: confidence interval; PDPH: postdural puncture headache; PONV: postoperative nausea and vomiting.

## Data Availability

The datasets used and/or analyzed during the current study are available from the corresponding author on reasonable request.

## Abbreviations

AOR: Adjusted odds ratio
CI: Confidence interval
COR: Crude odds ratio
CS: Caesarean section
HMIS: Health management information system
OMH: Orotta maternity hospital
OR: Odds ratio
PONV: Postoperative nausea and vomiting
PDPH: Postdural puncture headache
SA: Spinal anesthesia
WHO: World Health Organization
MOH: Ministry of Health
ACHS: Asmara College of Health Sciences.

## Supplementary Materials

Supplementary Materials The supplementary material is the questionnaire form used for data collection.
