# Supplementary material for: Maternal Satisfaction and Its Associated Factors towards Spinal Anesthesia for Caesarean Section: A Cross-Sectional Study in Two Eritrean Hospitals
Source: Anesthesiol Res Pract. 2020 Mar 21;2020:5025309. doi: 10.1155/2020/5025309 (PMC7114738; doi:10.1155/2020/5025309)
Supplement: Supplementary Materials — The supplementary material is the questionnaire form used for data collection. [file 5025309.f1.pdf]

Questionnaire Form

Data collection format prepared for maternal satisfaction and its associated factors towards spinal anesthesia for cesarean section.

NO.	A. Demographic status of the patient		Skip to
**1**	Hospital	Sembel……………...1<br>Orotta Maternity…… 2	
**2**	Age (years)		
**3**	Level of Education	Illiterate…………….…0<br>Primary ……………..1<br>Junior ...…………….2<br>Secondary...…………3<br>Tertiary……….…….4	
**4**	Employment	Employed …………..1<br>Unemployed ……......2	
**5**	Marital status	Married ……….…....1<br>Single ………..…....2	
	**B. Parturition history**		
**6**	Parity	Primipara……..…….1<br>Multipara…………....2	
**7**	Neonate birth weight	<3kg ………………..1<br>>3kg ………………..2	
**8**	APGAR Score in 10 min	10 …………………1<br><10 ………………..2	
	**C. Past medical, surgical and anesthetic experience**		
**9**	Does patient have any medical history?	Yes………………..1<br>No …..……………2	
**10**	Has patient received anesthesia before?	Yes ………………1<br>No ………………..2	--------→12
**11**	What technique?	GA ………………1<br>SA ……………….2	

**12**	Had patient complication from receiving anesthesia before?	Yes………………1<br>No………………...2	---------→14
**13**	What was the complication?	PONV……………1<br>Urinary retention…2<br>Pain………………3<br>Backache…………4<br>Others……………..5<br>(specify):__________	
	**D. Perioperative care**		
**14**	Did you have intra operative pain?	Yes…………………1<br>No ………………….2	
**15**	Did you have post-operative backache?	Yes………………….1<br>No…………………...2	
**16**	Did you have PONV?	Yes …………………1<br>No …………………..2	
**17**	Number of Attempts at puncture site?	<=2 trials……………1<br>>2trials……………...2	
**18**	Are you happy to take spinal anesthesia for same procedure C/S?	Yes…………………1<br>No…………………...2	----------→20
**19**	If answer to No. 18 is No why?	Fear of awareness…..1<br>Pain ………………...2<br>Nausea&vomiting..….3<br>PDPH ……………… 4<br>Others……………..5<br>(specify):__________	

20. Satisfaction questions

		**1**	**2**	**3**
No.	**Preoperative Assessment and Evaluation**			
1	Satisfaction with the amount of information given from the anesthesia practitioners about anesthesia			

2	Explanation about operation			
3	Explanation about your stay at OR			
4	Understandable information			
5	Sufficient explanation about the feeling after anesthesia			
6	Satisfaction with preoperative visit			
7	Satisfaction with information about postoperative complication			
No.	**Pain Therapy**			
8	Satisfaction with postoperative pain			
9	How you satisfied with the absence of pain at puncture site			
10	Satisfaction with absence of pain during operation			
No.	**Attention by the Anesthetist**			
11	Satisfaction of anaesthetist's attention to your complaints like pain and nausea			
12	Satisfaction with degree of anaesthetist's will to listen your questions			
13	Action according to your needs			
14	Anesthetist show understanding for your situation			
No.	**Anesthetist –patient relationship**			
15	Did the anesthetist take into account your privacy?			
16	Anesthetist's respect/politeness			
17	Anesthetist account on your cultural background?			
18	Satisfaction with the chance for your decision on type of anesthesia received			
No.	**Postoperative care**			
19	Satisfaction with anaesthetists postop visit			
20	Satisfaction with PONV treatment			
No.	**Quality care**			
21	Satisfaction with waiting time between your arrival at theatre and operation			
22	Satisfaction to receive same anesthetic again			
23	Degree of your confidence in the anesthesia practitioners			
24	Degree you find your anesthesia practitioners professional			
25	Recommendation of the anesthesia team to others in your family			

Key: 1= dissatisfied 2 = Neutral 3 = Satisfied
